# Radiochromic film dosimetry of rectal inhomogeneity and applicator attenuation in high dose rate brachytherapy of uterine cervix

**DOI:** 10.1120/jacmp.v13i1.3654

**Published:** 2012-01-05

**Authors:** Satish C. Uniyal, Umesh C. Naithani, Sunil D. Sharma, Anoop K. Srivastava

**Affiliations:** ^1^ Department of Radiology, Himalayan Institute of Medical Sciences HIHT University Jolly Grant Dehradun 248140 India; ^2^ Department of Physics HNB Garhwal Central University, Campus Pauri Pauri (Garhwal) Uttarakhand 246001 India; ^3^ Radiological Physics & Advisory Division Bhabha Atomic Research Centre, CT&CRS Building Anushaktinagar Mumbai 400094 India; ^4^ Cancer Research Institute HIHT University Jolly Grant Dehradun 248140 India

**Keywords:** brachytherapy, dosimetry, treatment planning, film, Monte Carlo

## Abstract

Heterogeneities existing in the patient during treatment are neglected, as the treated subject is considered homogeneous in most of the commercially‐available treatment planning systems (TPSs) used for high dose rate (HDR) brachytherapy. The choice of a suitable dosimeter for experimental dosimetry near the HDR source is crucial, mainly due to existence of steep dose gradients. The present work aimed to assess the effect of rectal air heterogeneity and applicator attenuation in the HDR Ir‐192 brachytherapy treatment of carcinoma uterine cervix by utilizing GAFCHROMIC EBT2 film dosimetry. The dose to rectal walls under the condition of rectal air heterogeneity was measured experimentally using EBT2 film in a rectal phantom, and the measurements were validated by the Monte Carlo (MC) simulations. The applicator attenuation was measured by EBT2 film for a commonly used stainless steel uterine tube in a homogeneous water equivalent phantom. The measured doses were compared with the TPS calculated values. In case of the air cavity, the measured dose at the closest rectal surface was 12.8% less than the TPS calculated value due to lack of back scattering, whereas at the farthest rectal surface, it was higher by 24.5% due to no attenuation. The magnitude of attenuation due to the metal applicator was measured as high as 2% when compared with the TPS calculation. The dose reduction at the nearest rectal surface due to the effect of rectal air has indicated a clinically favorable dose distribution within the rectum, whereas the shielding effect posed by the metallic applicator was found to be less significant. Mutual agreement of the measured doses with the MC calculated dose values confirmed the suitability of EBT2 film for clinical dosimetry in HDR brachytherapy.

PACS numbers: 87.53.Bn, 87.53.Jw, 87.56.bg, 87.55.Qr

## I. INTRODUCTION

High dose rate (HDR) brachytherapy using a Ir‐192 source has become an accepted technique to treat gynecological malignancies because the irradiation time is short enough to provide treatment on an outpatient basis.^(^ ^1^ ^)^ The commercially available treatment planning systems (TPS) employ dose calculation algorithms which do not account for the effect of heterogeneities present in the patient and the shielding effects posed by applicators used in the treatment. This implies a mismatch between the planned and real doses delivered to the tumor and organs at risk. Brachytherapy of uterine cervix carcinoma often results in high doses to surrounding structures, such as rectum and bladder. Therefore, it is important to quantify the dose delivered to these organs simulating the conditions of treatment. A specific case is late rectal toxicity, which depends on the total radiation dose received by the rectum, irradiated rectal volume, and the treatment dose rate.^(^ ^2^ ^–^ ^5^ ^)^ However, the incidence and amount of subsequent rectal complications mainly depend on the total radiation dose delivered to the rectum.^(^ ^6^ ^)^

The dosimetric method used in intracavitary brachytherapy significantly affects the doses to the target volume and the organs at risk. Conventionally, dose specification for the cervix treatment has been carried out in terms of doses at some specified points. The International Commission on Radiation Units and Measurements (ICRU) advocated a standardized system of dose reporting using critical organ doses at specified reference points to evaluate rectal complications.^(^ ^7^ ^)^ It is a common practice to limit the total rectal ICRU doses to 70 Gy, unless it would compromise tumor control. However, whether the rectal point indicates an optimal point for evaluation of rectal complications is controversial, because of inadequate visualization of the whole rectum in orthogonal radiographs. The development of 3D image‐based treatment planning in brachytherapy has significantly improved the 3D assessment of dose‐volume relations.

Dose specification guidelines for 3D imaging have been proposed by the Gynecological GEC, European Society for Therapeutic Radiology and Oncology (GEC‐ESTRO).^(^ ^8^ ^)^ Accordingly, the minimum dose to the most irradiated rectal tissue volume adjacent to the applicator ($D_{0.1\text{cc}}$, $D_{1\text{cc}}$, $D_{2\text{cc}}$ for 0.1, 1 and $2\;{\text{cm}}^{3}$) is recommended for reporting. A general GEC‐ESTRO recommended rectal dose constrain for $D_{2\text{cc}}$ is 70 ${\text{Gy}}_{\text{EQD}2}$, where EQD2 is the total biologically equivalent dose in 2 Gy fractions.

In addition to the dosimetric method used, the dose distribution in the anatomy of interest may depend on composition of its organs at the time of treatment. As a clinical experience, we have observed the presence of rectal air in the CT images of most of the randomly selected patients undergoing intracavitary treatment, which may perturb the rectal dose pattern. Because of the organ's proximity to sources during brachytherapy treatment of cervix, a need exists to experimentally ensure the effect of rectal air on different points located on the rectum. Another important factor that needs consideration is the material of the applicator used in the treatment, which is not water equivalent as required by the TPS and, consequently, may add to the existing heterogeneity in the patient. A number of experimental and Monte Carlo (MC) studies have been reported in literature demonstrating the effect of inhomogeneity in brachytherapy treatments^(^ ^9^ ^–^ ^12^ ^)^ and the shielding effects created by vaginal applicators.^(^ ^13^ ^–^ ^14^ ^)^

The choice of a suitable dosimeter for experimental dosimetry near the HDR source is crucial mainly due to existence of steep dose gradients. The important detector requirements for brachytherapy dosimetry are high spatial resolution, energy independency, tissue equivalency, and convenience of use.^(^ ^15^ ^,^ ^19^ ^)^ The recently introduced GAFCHROMIC EBT2 film (ISP Technologies, USA) meets the requirement of high spatial resolution with small detecting volume for near field dosimetry in brachytherapy. The variation of dose along the film is expressed in more detail than at few discrete positions, as in the cases of diode and thermoluminescent dosimeters. Although the EBT2 film exhibits a slightly nonlinear dose response, it is nearly tissue equivalent and energy independent for energies of photon beams used in radiation therapy. ^(^ ^16^ ^)^ The calibration and dose measuring techniques employing the EBT2 film dosimetry are relatively easy and results are quick.^(^ ^17^ ^)^

In this paper, we present a method employing the EBT2 film for assessing the effects of inhomogeneity created due to rectal air and metal applicator in the HDR brachytherapy treatment of carcinoma uterine cervix. The experimental results were verified using Monte Carlo simulations and compared with the TPS calculated dose values.

## II. MATERIALS AND METHODS

### A. HDR unit and treatment planning system (TPS)

All the studies were carried out with the microSelectron HDR v2 remote afterloading brachytherapy unit (Nucletron International, The Netherlands). The mHDR‐v2 Ir‐192 source available in this remote afterloader has an active length of 3.6 mm and active diameter of 0.65 mm. The active source is contained in a stainless steel capsule of external diameter 0.9 mm and length 4.5 mm. A stainless steel cable is laser welded to one end of the source capsule to position the source into the treatment applicator at required locations with the help of a stepping motor. A CT simulator networked 3D treatment planning system, the Oncentra MasterPlan version 3.3 (Nucletron International, The Netherlands), which uses the AAPM TG‐43 formalism^(^ ^18^ ^–^ ^19^ ^)^ for dose calculation, is used as a dedicated TPS with this HDR unit at our center.

### B. GAFCHROMIC EBT2 film dosimetry

The GAFCHROMIC EBT2 dosimetry film QD+  (ISP Technologies, Lot Number F020609) is a self‐developing radiochromic film that does not require chemical/physical processing. According to the manufacturer, the photon response of the EBT2 film is nearly energy independent from 50 keV to MV range, which clearly includes energies emitted by the HDR Ir‐192 source. The film has an improved uniformity and high sensitivity which can be used in the dose range of 1 cGy–10 Gy in the red color channel and even wider dose range up to 40 Gy in the green color channel. The most attractive feature of the EBT2 film to be used for HDR brachytherapy is its high spatial resolution required to assess doses in steep gradient regions about the source. Furthermore, its overall effective atomic number ($Z_{\text{eff}}$) is 6.84, which makes it near tissue equivalent ($Z_{\text{eff}}$ of water is 7.42). The active part of the film is a single sensitive layer of thickness about 30 μm with a thin topcoat made on a clear 175 μm thick polyester substrate. The active layer is covered by a 50 μm thick polyester overlaminate with an adhesive layer of thickness 25 μm and, hence, the total film thickness is about 285 μm.^(^ ^20^ ^)^

The film was calibrated in a 6 MV photon beam produced by a medical electron linear accelerator (Siemens Primus M) in the dose range of 25–700 cGy. For this purpose, the film samples of size$4\;\times \;4\;{\text{cm}}^{2}$ were positioned at 10 cm depth in a $30\;\times \;30\;\times \;30\;{\text{cm}}^{3}$ polymethyl methacrylate (PMMA) phantom. The PMMA phantom was positioned at a source‐to‐surface distance of 100 cm and irradiated using $10\;\times \;10\;{\text{cm}}^{2}$ field. The exposed film samples were analyzed 48 hours after irradiation in order to ensure optimum growth of optical density. Each film sample was scanned in landscape orientation and in red color channel mode of Epson Expression 10000 XL flatbed scanner. The optical density (OD) of each pixel in the central $2\;\times \;2\;{\text{cm}}^{2}$ region of the film was measured and the mean optical density (MOD) was then calculated for each calibration film. A dose response curve for EBT2 film was plotted between MOD and corresponding value of the dose and a fit equation was obtained.

#### B.1 Experimental arrangement

**Figure 1:**
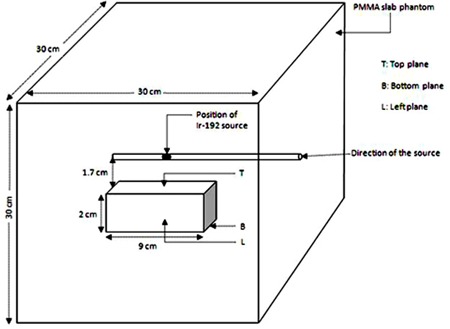
Schematic diagram of the experimental arrangement used for rectal dose measurement during high dose rate intracavitary brachytherapy.

The dose measurements were carried out in a specially designed and locally fabricated PMMA rectal phantom. PMMA was chosen as the phantom material because of its local availability, low cost, and ease of machining. Further, it is near tissue equivalent ($Z_{\text{eff}}=6.5$) and also equivalent to the EBT2 film, the dosimeter used in the present work. The phantom is designed to simulate a rectal cavity of a patient undergoing intracavitary brachytherapy. For this purpose, the volumes of rectal cavities of ten randomly selected patients were measured from the TPS, and the average volume was chosen as the volume of the rectal cavity in the phantom. The phantom is a cube of dimensions of $30\;\times \;30\;\times \;30\;{\text{cm}}^{3}$ comprising various slabs of PMMA. To simulate the inhomogeneity created by the rectal air, a rectum simulating cuboid cavity of dimensions $9\;\times \;2\;\times \;2\;{\text{cm}}^{3}$ was created at the center of the cubical phantom. For convenience, the simulated rectum cavity was assumed as a cuboid, although the real rectal cavity looks like an elliptical cylinder. Because the HDR source has a steep dose gradient and the shape and size of the rectum varies from patient to patient, the errors resulting from deviations from the actual geometry are small and irrelevant for the purpose of this work. A slab containing a groove for positioning a plastic catheter into it was placed above the rectal cavity such that the groove runs parallel and normally above the central length of the cavity. The source position into the applicator was at 1.7 cm away from the nearest surface of the cavity. In real cases, this distance depends on the individual patient and also on the selected CT slice. For the purpose of this study, an average value of 1.7 cm was obtained from the sample of randomly selected patients, as mentioned above. A schematic diagram of the experimental arrangement is shown in Fig. 1. To measure doses in a homogeneous medium (when there is no air in the rectum), a PMMA cuboid of dimensions $9\;\times \;2\;\times \;2\;{\text{cm}}^{3}$ was inserted into the cavity.

#### B.2 Experimental and TPS dosimetry

To acquire the confidence in EBT2 film dosimetry of Ir‐192 source, the relative depth dose (RDD) at different distances along the transverse plane of the source was experimentally determined in a $30\;\times \;30\;\times \;30\;{\text{cm}}^{3}$ PMMA phantom using this film. The RDD is defined as the ratio of dose rate at a given radial distance (r) to the dose rate at the reference distance ($r_{0}=1\text{cm}$) along the transverse axis from the center of the source. Further, the relative depth dose in this PMMA phantom was also calculated using the treatment planning system, and the two values of RDD so obtained were compared.

For determining the dose values at points of interest in the rectal phantom, EBT2 film samples were precisely cut into required sizes in order to fit at the top (T), bottom (B), left (L), and right (R) (not shown in the figure) surfaces of the air cavity created into the phantom. A dwell position to drive the source at a point normally above the geometric center of the cavity was programmed. The films were irradiated using a dwell time to deliver a dose to the films within the range 25–700 cGy. For dose measurements in the homogeneous tissue equivalent rectal cavity, a similar film configuration was used with the PMMA cuboid placed into the empty cavity. Each exposed film was scanned in an identical way as during the calibration, and their images were acquired. The images were digitized and their pixel values along the longitudinal and transverse bisectors of the film were obtained. The optical density at a point was calculated by using the pixel value of the point and mean background pixel value. Optical densities found in each case were further converted into absorbed doses by applying the previously obtained fit equation. The dose at the center of each surface of the cavity was obtained by averaging the doses at four pixels around the center point. The final values of dose were taken from an average of five measurements at the point and converted into dose to water by using a dose conversion factor from PMMA to water.^(^ ^21^ ^)^

The effect of applicator attenuation was assessed by measuring the dose from the HDR Ir‐192 source placed in a commonly used intrauterine tube (Nucletron vaginal applicator set part # 084.350) made up of stainless steel (AISI 316L, density $8.02\;g/{\text{cm}}^{3}$) having an outer diameter of 3.2 mm and a wall thickness of 0.6 mm. For this purpose, the applicator was positioned in a homogeneous PMMA phantom, and the dose at different radial distances along the transverse axis of the source was measured using GAFCHROMIC EBT2 film. The dose in this homogeneous PMMA phantom was also calculated using the TPS, which does not assume the presence of the applicator, and the results were compared with the corresponding values of the dose measured using EBT2 film.

### C. Monte Carlo simulation

The experimental results obtained in the present study were further validated using Monte Carlo simulations. For this purpose, the Monte Carlo code system EGSnrc version 4 was used.^(^ ^22^ ^–^ ^24^ ^)^ The EGSnrc code is a modified version of EGS4 code, in which changes have been introduced mostly to the algorithm and physics, to improve the accuracy of dose calculation. In this code, the history of primary and secondary photons, electrons, and positrons can be followed, and user defined parameters such as dose, energy deposition, and interaction counts are recorded.

The geometry modeled was the microSelectron HDR Ir‐192 source positioned at the center of a $30\;\times \;30\;\times \;30\;{\text{cm}}^{3}$ cube of water. The water density used in this simulation was $0.997\;\text{gm}/{\text{cm}}^{3}$ at 22°C. The geometric detail and composition of the HDR Ir‐192 (new design) source model used in this study were taken from the published studies.^(^ ^25^ ^)^ The stainless steel cable laser welded to one end of the source has a diameter of 0.7 mm. The cable was assumed to be 5 cm in length for the simulation, to take into account the backscatter contribution from the cable. The HDR Ir‐192 source emits gamma photons and beta particles. The stainless steel encapsulation of the source shields most of the beta particles. The remaining beta particles are attenuated in the medium and have no possibility of reaching the rectal cavity. So, the interaction of beta particles emitted from the source was not considered in the simulation and only gamma ray photons have been simulated.^(^ ^26^ ^)^ To simulate the source geometry and rectal cavity experiment, the deposited energy was determined by tracing $10^{9}$ histories. Simulated photon interactions included pair production, photoelectric absorption, Compton and Rayleigh scattering, and production of K‐edge characteristic X‐rays. The photon energy spectra for the source and the cross‐sectional tables needed for the Monte Carlo simulations have been obtained from EPDL 97.^(^ ^27^ ^)^

Calculation of air kerma strength was made by separately simulating the Ir‐192 source at the center of a cubical volume of dimensions of $5\;\times \;5\times 5\;m^{3}$ consisting of air with density of $0.12\times 10^{-2}g/{\text{cm}}^{3}$. The number of histories simulated was $10^{9}$. The calculations were made at different distances from 1 to 100 cm along the transverse axis from the source center using scoring cells of different sizes ranging from $0.5\times 0.5\times 1.0\;{\text{mm}}^{3}$ at 1.0 cm to $2\times 2\times 1\;{\text{mm}}^{3}$ at 100 cm in order to increase the deposition of energy. The dimensions of the scoring cells were chosen to avoid volume averaging errors due to the finite size of the cells.

Monte Carlo simulation was also carried out to calculate the absorbed dose at the points of interest mentioned in the rectal cavity experiment with and without assuming the air cavity. The simulations were performed three times to reduce the statistical uncertainty in dose calculation. The number of photon histories simulated in each case was $10^{9}$. The source and dose‐scoring cells were assumed within a water phantom of dimensions $30\;\times \;30\;\times \;30\;{\text{cm}}^{3}$. The sizes of scoring volumes were defined according to their distances from the source to make the number of interactions occurring within the volume sufficiently large, while minimizing the computation time and statistical uncertainty of the estimated dose response.

## III. RESULTS & DISCUSSION

### A. Film calibration and RDD

**Figure 2:**
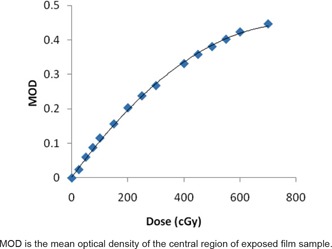
Dose response calibration curve of GAFCHROMIC EBT2 film in 6 MV X‐rays.

Figure 2 shows the dose response curve of GAFCHROMIC EBT2 film in 6 MV X‐rays which is a plot between MOD and corresponding value of the dose. This curve is nonlinear in nature. Equation (1) is a mathematical fit of the experimental data, which was obtained for subsequent determination of the absorbed dose from the measured optical density:

(1) $$D\;(\text{cGy})=2753\;{\left(\text{OD}\right)}^{2}+451.4\;(\text{OD})$$

where *D* is absorbed dose and *OD* is the optical density of the film.

The combined standard uncertainty in obtaining dose response calibration of EBT2 film is 3.2% (coverage factor, k=2) which is calculated by combining type A and type B uncertainties. This includes uncertainties in the dose rate calibration of the 6 MV X‐rays, film nonuniformity and film scanner reproducibility.

The values of relative depth dose measured with the EBT2 film and those calculated using brachytherapy TPS are presented in Table 1. The film‐measured and TPS‐calculated values of RDD are in good agreement with each other. Further, the EBT2 film‐measured and TPS‐calculated dose values were also found in good agreement with each other at distances less than 3 cm from the source, which include most of the clinically relevant distances. Beyond this depth, a higher discrepancy between the measured and calculated doses was noticed. The reason for this difference in EBT2 measured and TPS calculated doses for distances larger than 3 cm could be extremely low doses at larger depths and limited film response at these doses. The agreement between measured and TPS calculated dose values support the use of 6 MV X‐ray beam for film calibration and also confirm the suitability of EBT2 film dosimetry for patient‐simulating studies in brachytherapy. The combined standard uncertainty in determining dose to a point in the PMMA phantom by EBT2 film is estimated to be 3.8% (coverage factor, k=2).

**Table 1:** Experimentally‐measured and TPS‐calculated values of relative depth dose for the HDR Ir‐192 brachytherapy source.

	*Relative Depth Dose (RDD)*	
*Radial Distance r (cm)*	*Measured Using EBT2*	*Calculated Using TPS*
1.0	1.000	1.000
1.5	0.433	0.450
2.0	0.244	0.252
2.5	0.156	0.160
3.0	0.109	0.114
3.5	0.079	0.084
4.0	0.057	0.061
4.5	0.050	0.053
5.0	0.035	0.038

### B. Dose to rectum in homogeneous and heterogeneous conditions

**Figure 3:**
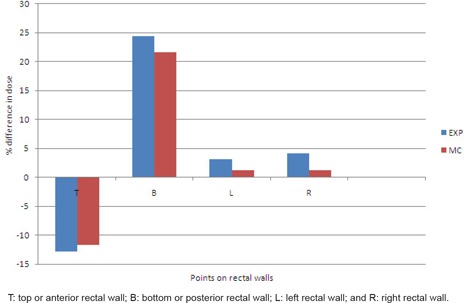
Bar chart of percentage difference in dose at rectal walls determined considering medium‐filled rectum and air‐filled rectum.

The doses at the points of interest in the phantom were determined with experimental and MC methods. The statistical uncertainties associated with the Monte Carlo estimated dose values were within ± 0.5%. The percentage difference between the dose in the case of medium‐filled rectal cavity and the dose in the case of air‐filled rectal cavity at top, bottom, left, and side rectal walls are shown in Fig. 3. The percentage difference in experimentally determined dose values as well as Monte Carlo calculated dose values are presented in this figure. The bar chart clearly indicates that the presence of rectal air produces significant differences in doses, mainly at the top and bottom planes of the cavity. For the top plane, which represents the anterior rectal wall, the negative direction of the bar shows that the dose is lower in the case of empty rectum than that for a homogeneous rectum. Numerically, the difference in dose determined by experimental method at the top plane is 12.8%, while this difference in Monte Carlo determined dose is 11.7%. The reduction in dose in the case of the empty rectum is attributed to the absence of a scattering medium in the rectal volume. At the bottom plane, which represents the posterior rectal wall, the film‐measured and MC‐calculated doses were found significantly higher for the case of air‐filled rectal cavity. The percentage difference in measured and MC‐calculated dose values at the bottom plane for empty and filled rectum is 24.5% and 21.7% respectively. The obvious reason for the higher doses in case of the empty cavity is the relatively less attenuation of photons in the air. For the left and right planes of the rectal cavities, the doses were found relatively higher in the case of empty rectal cavity. It is worth mentioning here that experimentally‐measured and MC‐calculated dose values at all the points were in good agreement. The significant difference in measured dose values and TPS calculated dose values at rectal walls under rectal air inhomogeneity indicates that the TPS should have dose calculation algorithm which should give the real dose values at the points of interests under the inhomogeneous conditions.

The experimental and MC results found in this study exist close to the values reported by Kwan et al.^(^ ^9^ ^)^ in a similar but methodologically different work, in which the effect of rectal air on the rectal dose was demonstrated in the treatment of prostate cancer by using MOSFET detectors and GEANT4 Monte Carlo simulation. The results of the present work are also comparable to the results of the study by Raina et al.^(^ ^10^ ^)^ which demonstrated the difference between the measured and planned doses due to the lack of scatter. In Raina's experimental study, the dose in the absence of a scattering medium was found to differ by up to 15% from that calculated by a brachytherapy treatment planning system.

Due to the presence of rectal air, the TPS overestimates the dose at and in the proximity of the anterior rectal wall, which is a region of interest in clinical treatment planning. The occurrence of rectal injury is shown to be correlated with certain specified point doses in the rectum.^(^ ^28^ ^–^ ^30^ ^)^ The ICRU rectal point is defined as the most anterior portion of the rectum with maximum dose value. According to the GEC ESTRO recommendations, the assessment of late rectal toxicity is indicated by small rectal volumes (0.1, 1, 2 cc) irradiated to a high dose which are used in the dose‐volume histograms (DVH)‐based specification of rectal dose. Due to rapid dose fall‐off near the sources, the adverse effects from brachytherapy occur mainly in these limited volumes which are most irradiated and essentially exist in the close proximity of the anterior rectal wall. The rectal air‐induced dosimetric changes across the rectal cavity are useful in achieving favorable dose distribution in the rectum, particularly at the anterior rectal wall which is maximally irradiated and likely to receive complications. As the real dose to anterior rectal wall under the rectal air inhomogeneity is relatively less, escalated dose treatment can be prescribed which may result in better tumor control without increasing the late rectum complications. Considering the beneficial effect of rectal air inhomogeneity (as far as dose to anterior rectal wall is concerned), it may not be necessary to control the rectal filling during intracavitary brachytherapy treatments.

### C. Applicator attenuation

Table 2 presents the GAFCHROMIC EBT2 film‐measured reduced dose values due to applicator attenuation as a percentage of the TPS calculated dose at different radial distances, along with the published values of Ye et al.^(^ ^13^ ^)^ The EBT2 film‐measured values and the published values of Ye et al. are in good agreement. It is also observed from the data in this table that the uterine tube offers about 2% of reduction along the transverse plane of the source, and the TPS neglects the applicator attenuation and overestimates the dose. At oblique angles, the photon beam travels even a longer path within the applicator, and hence may result in further dose reduction.

**Table 2:** Comparison of the EBT2 film measured and published values of dose reduction by the applicator expressed as percentage of the dose of a bare source measured along its transverse axis.

	*Reduced Dose Value (%) Due to Applicator Attenuation*			
		*Ye et al.* ^(^ ^13^ ^)^		
*Transverse Distance r (cm)*	*EBT2 Film‐measured (Present Work)*	*Experimental*		*Monte Carlo*
1	98.0	98.4		98.6
2	98.2	98.5		98.7
3	98.4	98.5		98.7
4	98.3	98.6		98.8
5	98.6	98.4		98.5
6	98.7	98.7		98.5

## IV. CONCLUSIONS

The present study was to quantify the effect of treatment heterogeneities created due to the rectal air and applicator material in HDR brachytherapy of cervical malignancies, by utilizing the GAFCHROMIC EBT2 film dosimeter. The TPS used in the study is based on the AAPM TG‐43 formalism of dose calculation, in which the medium of measurement is considered to be homogeneous and water equivalent. Consequently, significant discrepancies exist between the planned and real treatment conditions as shown by the results of the study. While the effect is quite significant in the case of the air inhomogeneity due to the lack of full scatter, it is not so significant in the case of applicator attenuation. The experimental results indicate that the presence of rectal air is a desired condition, because the real dose to the anterior rectal wall is lower than the TPS‐calculated dose during the presence of rectal air. The agreement between measured and MC‐computed values showed the suitability of the EBT2 film dosimetry for patient simulating studies in brachytherapy. The same is also verified by the mutual agreement of film‐measured and TPS‐calculated values of RDD. This study confirms the feasibility of EBT2 film as a suitable detector for point dosimetry in HDR brachytherapy.

## ACKNOWLEDGMENTS

The authors would like to thank Dr. Y. S. Mayya, Head, Radiological Physics & Advisory Division, Bhabha Atomic Research Centre, Mumbai for permission to use the experimental facilities during this work. The authors would also like to thank Dr. Samuel D., Head, Department of Radiology and Dr. Sunil Saini, Director, Cancer Research Institute, HIHT University, Jolly Grant, Dehradun for their support in this work.
